# Significant associations between bone mineral density and vascular calcification in patients with different stages of chronic kidney disease

**DOI:** 10.1186/s12882-022-02955-9

**Published:** 2022-10-05

**Authors:** Jana Uhlinova, Anne Kuudeberg, Kaja Metsküla, Margus Lember, Mai Rosenberg

**Affiliations:** 1 Department of Internal Medicine, Institute of Clinical Medicine, University of Tartu, Puusepa str. 8, 50406 Tartu, Estonia; 2 Department of Internal Medicine, Tartu University Hospital, Tartu, Estonia; 3 Department of Anatomy, Institute of Pathological Anatomy and Forensic Medicine, University of Tartu, Tartu, Estonia; 4 Department of Immunology, Institute of Biomedicine and Translation Medicine, University of Tartu, Tartu, Estonia

**Keywords:** Abdominal aortic calcification, Ankle-brachial index, Bone mineral density, Chronic kidney disease, DEXA

## Abstract

**Introduction:**

Chronic kidney disease—mineral and bone disorders (CKD-MBD) is characterised by generalised vascular calcification (VC) and impaired bone health. We aimed to investigate the relationship between VC and bone mineral density (BMD) in CKD patients.

**Methods:**

We performed a cross-sectional study of patients with different stages of CKD. For assessment of VC of abdominal aorta lateral lumbar X-rays (Kauppila score), the ankle-brachial index (ABI) and echocardiography were used. Total body densitometry provided BMD.

**Results:**

Ninety patients (41% male, median age 64 years (range 29–87)) were included, of whom 41.1% had a Kauppila score > 1. Evidence of peripheral VC as measured by ABI was detected in 23.3% of cases. Lesions of the heart valves were found in 46.7% of patients. There was a significant association between high ABI and lesions of the heart valves. In the multivariate regression model to analyse the independent determinants of abdominal aorta calcification (AAC) and ABI, the BMD of the femoral neck was identified as significant for both (*p* = 0.001, *p* = 0.001). The total spine BMD was found to be significant for AAC (*p* = 0.001), and the BMD of spine L1-L4 and the ribs were found to be significant for ABI (*p* = 0.01, *p* = 0.002 respectively). In factorial regression analysis, where BMD was independent determinant, valvular calcification was significant for BMD of femur, femoral neck and total BMD. Age and tALP were inversely correlated with the BMD of femur and femoral neck.

**Conclusions:**

Our work highlighted clinically important relationships between VC and bone mineral density (BMD) in CKD patients. We detected inverse relationships between AAC, high ABI and BMD. Secondly, BMD at certain bone sites (femur, femoral neck) and total BMD were associated with important lesions of heart valves. Thirdly, a significant association between a high ABI and lesions of the heart valves. We believe that the results of our study will help in the planning of future research and in current clinical practice for the early diagnosis, further monitoring and management of CKD-MBD. Additionally, these results may have treatment implications on use of different CKD-MBD medications.

## Introduction

The prevalence of chronic kidney disease (CKD) is still growing, affecting about 10% of all populations worldwide, and the incidence of CKD-MBD (CKD-mineral and bone disorder) is increasing in parallel [1]. CKD-MBD causes cardiovascular disease with premature arteriosclerosis and accelerated vascular calcification (VC) which leads to generalized VC. CKD-MBD moreover, is characterised by impaired bone health caused by renal osteodystrophy and osteoporosis [2]. Additionally to age-related osteoporosis, renal osteodystrophy is a common disorder, characterised by a combination of reduced bone mineral density (BMD) due to abnormal bone turnover rate, and impaired bone tissue quality. Bone disease in CKD is associated not only with increased risk of fractures but also with increased VC, vascular stiffening and vascular senescence [2]. Altogether these mineral and bone disturbances lead to morbidity and premature mortality in CKD and end-stage kidney disease (ESKD) [3–5]. CKD-MBD is often progressive but typically remains clinically silent until stages G3b-G4 [6]. The measurement of femoral and lumbar BMD by dual-energy X-ray absorptiometry (DEXA) is a standard for diagnosis of osteoporosis in the general population [7–10] but it has limitations in CKD patients because of abdominal aorta and vertebral articular calcification. Because of these facts total body DEXA is more accurate method for measurement of BMD in CKD patients. However, from the clinical point of view and the diagnosis of osteoporosis, total body DEXA remains as an informative tool but not diagnostic.

Interestingly, researchers have found that bone mass at cortex-rich sites such as the femoral neck is more affected than trabecular bone, and these appear to be the most useful sites for predicting clinical outcomes in CKD patients [2]. Because of the limited data, there are yet no consensus decisions on the use of DEXA for measurement of BMD in CKD patients. In addition, there are gaps in knowledge in terms of the epidemiology, pathophysiology, diagnosis and treatment of patients with low BMD or VC. There are only a few studies focusing on the relationship between central and peripheral calcification in those with bone disease [5, 11–13]. We aimed to find out if there are significant associations between central or peripheral calcification and different DEXA parameters. We therefore studied the potential associations between VC and BMD in CKD patients with different stages using a range of non-invasive methods for detection of VC and total body DEXA for measurement of site-specific BMD.

## Subjects and methods

### Study subjects

The studied cohort included 168 consecutive patients with different stages of CKD from Nephrology department at Tartu University Hospital [14]. Ninety patients of the cohort were included into this study based on the availability of DEXA results. Inclusion criteria were age > 18 years, CKD diagnosis, regular observation by nephrologist.

The CKD diagnosis was defined via eGFR and albuminuria according to KDIGO guidelines [15, 16]. The Chronic Kidney Disease Epidemiology Collaboration (CKD-EPI) formula was used to assess kidney function. Using classification of CKD kidney function ranged from G1A3 – G5A3. The distribution of patients by stages was as follows: G1-G2 12 patients, G3a 15 patients, G3b 24 patients, G4 18 patients and G5 20 patients including 8 dialysis patients.

Participation was voluntary and, once fully matched to an individual, all data were anonymised. Signed informed consent was obtained from participants. The Ethics Review Committee on Human Research at the University of Tartu approved the study (approval no. 223/T-17). Clinical data were collected from the electronic medical records. All patients have treatment of CKD and its complications according to best practice international guidelines [1, 16].

### Vascular calcification

As recommended by the KDIGO working group [1] we used radiography of the abdominal aorta for detection of central VC and echocardiography for heart valve lesions. The ABI was used to assess peripheral VC.

#### Assessment of abdominal aorta calcification

In common with many previous studies, we used lateral lumbar X-rays to assess calcification of the abdominal aorta (AAC). We used the semi-quantitative scoring system, as described by Kauppila et al. [17]. This is an informative, non-invasive method to detect and measure the progression of VC events [18, 19]. The abdominal aorta alongside the first four lumbar vertebrae is divided into four segments using the midpoint of each intervertebral space as a boundary. Anterior and posterior aortic wall segments are evaluated separately. Calcific deposits are graded on a scale of 0–3 at each segment, as follows: zero—no calcific deposits; 1—small-scattered calcific deposits filling less than one-third of the aortic wall; 2—one-third to two-thirds of the aortic wall calcified; 3—at least two-thirds of the aortic wall calcified. The grades of the eight aortic segments are summated to yield the Kauppila calcification score, which ranges from zero to 24 points. An AAC value of 0 was defined as normal, 1 – 6 as moderate, and 7 – 24 as severe calcification. Two independent radiologists scored all lateral lumbar X-rays. Both observers were kept blind to the clinical and laboratory patient data.

#### Ankle-brachial index assessment

The ankle-brachial index (ABI) is defined by the ratio of ankle and brachial systolic pressure [20]. A simple, non-invasive, accurate tool to evaluate arterial stiffness and PAD, this method provides diagnostic and prognostic information when values are ≥ 1.3 or < 0.9 [21]. The systolic pressure in the foot was measured with an Atys Microflow Doppler ultrasound device and was assessed on the tibiae posterior and dorsalis pedis arteries as described in our previous study [14]. The measurement of blood pressure in both arms was performed mechanically. The ABI was calculated separately for each leg. The final ABI values were determined by taking the higher pressure of the two arteries in each foot, divided by the higher of the two brachial arterial systolic pressures. ABI measurement was done once in each patient after a 15-min rest period. Patients were assigned to one of three classifications: ABI < 0.9 in either foot, ≥ 0.9 to < 1.3 in both feet, and ≥ 1.3 in either foot.

#### Echocardiography for evaluation of cardiac structure and function

Experienced cardiologists at Tartu University Hospital performed echocardiography. The calcification and fibrosis of each heart valve was evaluated separately according to standard echocardiography methodology and classified as yes or no.

### Assessment of bone mineral density

BMD was assessed by total body DEXA using the Lunar Prodigy Advance device, GE Healthcare, which provides results for multiple individual sites. All scans were performed by certified operator and analysed by experienced rheumatologist.

BMD was measured as the ratio of grams per centimetre squared (g/cm^2^) and the NHANES III/Lunar population reference data was used to calculate T-scores relative to young healthy adults and age-matched Z-score [22].

### Laboratory data

Laboratory research was carried out at the United Laboratories of Tartu University Hospital. The following biochemical parameters were assessed: serum albumin (S-Alb, g/L), serum C-reactive protein (S-CRP, mg/L), serum creatinine (S-Crea, µmol/L), serum urea (S-Urea, mmol/L), serum total calcium (S-Ca, mmol/L), serum ionized calcium (S-i-Ca, mmol/L), serum phosphate (S-Pi, mmol/L), serum uric acid (S-UA, mmol/L), serum total cholesterol (S-CHL, mmol/L), serum triglyceride (S-TG, mmol/L). Also, serum haemoglobin (S-Hb, g/L) was assessed.

Kinetic colorimetric assay for measurement of serum total alkaline phosphatase (S-tALP, normal range 35–128 iu/L) was used.

Serum 25(OH)D (S-vit D (25 OH), > 50 nmol/L) and intact parathyroid hormone (iPTH, 1.6–6.9 pmol/L) was assessed by electrochemiluminescence immunoassay and Elecsys kit (Roche) was used.

For evaluating of intact fibroblast growth factor 23 (iFGF-23, U/mL), serum was separated from peripheral venous blood samples, stored at –80 °C, and analysed by ELISA method (Millipore Corporation, Billerica, MA, USA). The normal range of iFGF-23 according to the description of the method is < 114 U/mL.

### Statistical analysis

Continuous variables were described by means and standard deviations or by median and range, as appropriate. Numbers and percentages were used for categorical variables. Pair-wise comparisons were done with the Mann–Whitney U test. BMD, AAC and ABI were treated as continuous and lesions of heart valves as dichotomous variables. Multivariate analyses were conducted using the Spearman rank order correlation method. Generalized linear models were performed to determine significant associations between ABI, Kauppila score and other variables. To avoid collinearity the stepwise backward elimination was used, beginning with the variable with the highest *p*-value. Factorial regression analysis was performed to determine significant associations between DEXA and other variables.

The results of the linear multivariate regression analysis were controlled with Bonferroni test and models determinant (R^2^) was 0.6. All analyses were performed using Statistica (version 13.5) software. Tests were two-sided and a *p* value of 0.05 was considered as statistically significant.

## Results

### Baseline characteristics

Demographic and clinical characteristics of investigated ninety patients (41% male) with a median age of 64 years (range 29–87) are presented in Table 1.

**Table 1:** Clinical characteristics of patients

Parameters	Median (range) or frequency (%)
Age (years)	64 (29–87)
Gender (male)	37 (41%)
BMI (kg/m^2^)	28 (16–49)
*Cause of CKD*	
DM (%)	28.9
HT (%)	27.8
Interstitial nephritis (%)	15.5
GN (%)	13.3
Other (%)^a^	14.5
Smoking (%)	6.7
Kauppila score > 1 (N)	37 (41.1%)
ABI ≥ 1.3 (N)	21 (23.3%)
ABI < 0.9 (N)	16 (17.8%)
Heart valve calcinosis/fibrosis (N)	42 (46.7%)

*BMI* Body mass index, *DM* Diabetes mellitus, *HT* Hypertension, *GN* Glomerulonephritis, *ABI* Ankle-brachial index

^a^Polycystic kidney disease, systemic vasculitis, amyloidosis

Diabetes mellitus and hypertension were the commonest causes of CKD (28.9% and 27.8%, respectively). There was a relatively low percentage of current smokers in the cohort (6.7%). There were significantly more smokers among males (13.5% vs 1.9% *p* = 0.03). A Kauppila score > 1 was detected in 41.1% of cases. Evidence of peripheral VC as measured by ABI was detected in 23.3% of cases. Valvular calcification was found in 46.7% of patients.

Laboratory and BMD data are displayed in Tables 2 and 3. The mean creatinine and eGFR were 261.7 µmol/L and 35.2 ml/min, respectively. The majority of patients had good mineral control, with stable haemoglobin and cholesterol levels, probably due to sufficient and timely drug treatment.

**Table 2:** Biochemical characteristics

*Serum markers*	Mean ± SD	Median (range)	Normal range
S-Crea (µmol/L)	261.7 ± 249.4	150.5 (51–1036)	45–104
eGFR (ml/min)	35.2 ± 22.8	33.5 (2–106)	> 90
S-Urea (mmol/L)	14.1 ± 7.9	11.9 (3.9–36.0)	< 8.1
S-Hb (g/L)	123.8 ± 18.2	120 (86–164)	121–170
S-CRP (mg/L)	5.0 ± 11.8	2 (1–107)	< 5
S-Alb (g/L)	41.7 ± 4.7	42 (27–54)	35–52
S-Ca (mmol/L)	2.33 ± 0.2	2.3 (1.8–2.8)	2.20–2.55
S-Pi (mmol/L)	1.36 ± 0.5	1.2 (0.7–3.4)	0.81–1.45
iPTH (pmol/L)	23.7 ± 43.0	10.5 (1.4–320.4)	1.6–6.9
S-tALP (iu/L)	113.1 ± 162.7	84 (0.9–1390)	35–129
S-CHL (mmol/L)	5.5 ± 1.3	5.3 (3.2–9.8)	< 5.5
S-TG (mmol/L)	1.89 ± 1.14	1.7 (0.5–5.1)	< 2.0
S-UA (mmol/L)	406.1 ± 92.4	395 (253–718)	143–417
S-vit D (25 OH) (nmol/L)	58.7 ± 27.9	48.5 (14–144)	> 50
FGF-23 (U/mL)	266.0 ± 602.2	51 (10–2400)	< 114

*S-Crea* Serum creatinine, *eGFR* estimated glomerular filtration ratio, *S-Urea* Serum urea, *S-Hb* Serum haemoglobin, *S-CRP* Serum c-reactive protein, *S-Alb* Serum albumin, *S-Ca* Serum calcium, *S-Pi* Serum phosphate, *PTH* Parathormone, *S-tALP* Serum total alkaline phosphatase, *S-CHL* Serum cholesterol, *S-TG* Serum triglycerides, *S-UA* Serum uric acid, *S-vit. D* Serum vitamin D, *FGF-23* Fibroblast growth factor 23

**Table 3:** Bone mineral density parameters

*Bone mineral density*	Mean ± SD	Median (range)
BMD total (g/cm^2^)	1.18 ± 0.14	1.2 (0.80—1.44)
BMD femur total (g/cm^2^)	0.96 ± 0.16	0.9 (0.55—1.27)
BMD femur neck (g/cm^2^)	0.88 ± 0.15	0.8 (0.55—1.21)
BMD total spine (g/cm^2^)	1.13 ± 0.18	1.1 (0.77—1.68)
BMD spine L1-L4 (g/cm^2^)	1.18 ± 0.22	1.1 (0.71—1.84)
BMD pelvis (g/cm^2^)	1.12 ± 0.15	1.1 (0.67—1.46)
BMD ribs (g/cm^2^)	0.70 ± 0.09	0.7 (0.51—1.0)
T-score total	0.25 ± 1.72	0.4 (-4.8—3.2)
Z-score total	0.90 ± 1.74	1.0 (-4.8—4.3)
T-score femur total	-0.56 ± 1.25	-0.6 (-3.8—2.1)
Z-score femur total	0.33 ± 1.12	0.3 (-3.9—1.3)
T-score femoral neck	-1.12 ± 1.12	-1.1 (-3.9—1.3)
Z-score femoral neck	0.03 ± 1.14	0.1 (-3.5—2.7)
T-score spine L1-L4	0.02 ± 1.83	-0.2 (-3.9—5.2)
Z-score spine L1-L4	0.89 ± 1.93	0.7 (-3.5—5.7)

*BMD* Bone mineral density

### Bone mineral density

Spearman Rank correlation analysis revealed statistically significant correlation between BMD of the femoral neck and age. BMD of femur, total spine, spine L1-L4, ribs, pelvis and total BMD inversely correlated with tALP level.

An inverse correlation was found between iPTH level and BMD of femoral neck, total spine, spine L1-L4, ribs and total BMD. A positive correlation was found between BMD of femur, femoral neck, ribs, pelvis and haemoglobin level. Similarly, a positive correlation was found between eGFR and BMD of the ribs (*p* = 0.003) and pelvis (*p* = 0.04) only. Statistically significant correlations between BMD and S-Ca or S-Pi were found only in the ribs.

In summary, the associations between BMD and tALP, iPTH, haemoglobin level were found. The ribs were the most common site at which correlations with biochemical parameters were found.

In the multivariate factorial regression model to analyse the independent determinants of AAC and ABI, the BMD of the femoral neck was identified to be significant for both. Also the total spine BMD, and the BMD at L1-L4 and of the ribs were found to be significantly correlated to AAC and ABI respectively (Table 4).

**Table 4:** Multivariate factorial regression analysis with variables entering the equation as correlates of BMD measurements with Kauppila score and ABI as dependent variables (only statistically significant results are marked in table)

	*R*^*2*^	Coefficient	95% CI	*p*-value
***Kauppila score***	0.53			
BMD femoral neck		-33.5	-49.2—-17.7	0.001
BMD total spine		-25.1	-40.1—-10.0	0.001
***ABI***	0.39			
BMD femoral neck		-2.21	-3.09- -1.33	0.001
BMD spine L1-L4		-0.77	-1.33-—0.21	0.01
BMD ribs		-1.69	-2.74- -0.65	0.002

*BMD* Bone mineral density, *ABI* Ankle-brachial index

In the factorial regression analysis, where BMD was independent determinant, valvular calcification was significant for BMD of femur, femoral neck and total BMD. Age and tALP were inversely correlated with the BMD of femur and femoral neck (Table 5).

**Table 5:** Factorial regression analysis, with BMD as independent variable; heart valve calcinosis, age, S-tALP as dependent variables

	*R*^*2*^	Coefficient	95% CI	*p*-value
***BMD femur total***	0.43			
Heart valve calcinosis		-2.48	-4.50—-0.46	**0.01**
Age		-0.03	-0.06—-0.01	**0.02**
S-tALP		-0.02	-0.04—-0.01	**0.01**
***BMD femoral neck***	0.49			
Heart valve calcinosis		-2.60	-4.28—-0.92	**0.003**
Age		-0.03	-0.06—-0.01	**0.001**
S-tALP		-0.02	-0.03—-0.01	**0.001**
***BMD total***	0.41			
Heart valve calcinosis		-1.88	-3.69—-0.06	**0.04**
Age		-0.02	-0.04—0.01	0.08
S-tALP		-0.01	-0.03—0.01	0.1

Statistically significant *p*-value < 0.05 and in bold

*BMD* Bone mineral density, *S-tALP* Serum total alkaline phosphatase

### Vascular calcification

There were 41.1% of patients with a Kauppila score > 1, 46.7% with valvular calcification, 23.3% with an ABI ≥ 1.3 and 17.8% with an ABI < 0.9. Positive correlations between AAC and age (*p* = 0.001), total calcium (*p* = 0.004) and cholesterol (*p* = 0.01) were found by Spearman Rank analysis.

In a linear multivariate regression analysis among predictive variables DM (*p* = 0.002), tALP (*p* = 0.001) and heart valve lesions (*p* = 0.002) were significantly associated with high ABI.

## Discussion

Our study was designed to evaluate associations between VC and BMD in CKD patients with different stages of disease using three non-invasive methods for assessment central and peripheral VC and total body DEXA for measurement of BMD in different skeletal sites. Our work highlighted clinically important relationships between VC and BMD in CKD patients. The study results showed significant associations between the Kauppila score and BMD of the femoral neck and total spine; and between ABI and BMD of the femoral neck, spine L1-L4, and ribs. Heart valve lesions were significantly associated with BMD of the femur, femoral neck and total body BMD. In some previous works, potential associations between VC and BMD were sought using different methods for evaluation of VC and BMD, but the results of these studies are somewhat controversial. For example, Toussaint et al. showed an inverse association between VC of the superficial femoral arteries as measured by CT and femoral BMD measured by DEXA [19]. However, some other researchers were unable to find any correlation between VC and BMD parameters [11, 12].

Nevertheless, our results are supported by previous investigations showing not only the inverse correlation between BMD and VC, but also that the progression of VC is accompanied by greater bone loss [13, 23]. A longitudinal study of older women has shown that moderate aorta calcification is been linked to increased fracture risk [24]. A similar interplay between the two pathological processes of VC and bone disorders was also described in some experimental studies [25, 26].

Bone biopsy is a gold standard for evaluation of bone tissue quality: turnover, mineralization and volume. Bone biopsy studies have provided important insights into the patterns of renal osteodystrophy, but despite of being recommended by KDIGO guidelines the utility of this invasive method in routine clinical practice is limited [1, 27].

There is a quite large number of non-invasive approaches to assess bone quality in kidney-related bone disease. Among these alongside conventional quantitative computed tomography (QCT), high-resolution peripheral QCT (HRpQCT) and micro magnetic resonance imaging (MRI), which give information about bone microarchitecture. The most accessible low-dose radiation imaging modality used in routine practice for measurement of bone mass and density is DEXA [27, 28]. Although different measurement modalities give a different information of bone tissue quality, recent study by Carvalho et al. has demonstrated a significant association between BMD by DEXA and bone histomorphometry data by bone biopsy, supporting the usefulness of DEXA in CKD patients [29]. In addition, new KDIGO guidelines changed the paradigm of the use of DEXA BMD as predictive tool for fractures. Based on results of four prospective cohort studies the Work Group concluded that DEXA BMD assessment is reasonable if a low or declining BMD will lead to additional interventions to reduce falls or use osteoporosis medications [1].

In spite of these limitations, DEXA is a good method to assess BMD and predict the risk of fractures in CKD patients [27, 30, 31].

DEXA is the clinical standard to measure bone mass and fracture risk in the general population, but it has limitations, especially in CKD patients. Central DEXA is generally recommended for the diagnosis of osteoporosis [8, 32] but in CKD patients, the BMD of the spine may be overestimated because of aortic calcification. In this context, total body DEXA is probably the more appropriate method for assessment of skeletal mineral status [2, 33]. Obviously, the optimal choice of DEXA type depends mainly on its purpose and in clinical practice should be chosen individually. In our study, we used total body DEXA not central DEXA.

Additionally, in the context of recent researches of CKD-MBD a site-specific BMD assessment is very important. The skeleton of humans is composed of cortical and trabecular bone. Trabecular bone, which makes up two-thirds of total bone surfaces, shows greater metabolic activity than cortical bone [34]. The proportions of cortical and trabecular bone vary between different skeletal sites: for example vertebrae are trabecular rich sites, however in long bones, the diaphysis consists of mainly cortical bone and metaphysis and epiphysis have mix structure of trabecular and cortical tissue. In contrast to general population, in CKD patient’s loss of cortical bone is more severe than loss of trabecular bone. Interestingly, researchers have found that bone mass at cortex-rich sites such as the femoral neck is more affected than trabecular bone, and these appear to be the most useful sites for predicting clinical outcomes in CKD patients [29, 35]. While DEXA cannot provide details regarding the relative proportions of the cortical and trabecular bone or distinguish between different types of renal osteodystrophy, it is still a good method for evaluation BMD in CKD patients. Furthermore, we confirmed the high incidence of central and peripheral VC in the CKD population demonstrated in our previous study. A significant association between central and peripheral VC in CKD patients was also found [14]. The current study revealed an additional significant association between high ABI and heart valve calcinosis/fibrosis. This important finding leads us to reconsider the place of ABI. Although still not recommended by current clinical guidelines we believe its role has been underestimated and that this is an effective and simple method to evaluate VC [36]. Moreover, a combined approach may give very good prognostic information, as studies from other groups have reported an increase in cardiovascular and all-cause mortality with pathologically high or low ABI, as well as with AAC, and heart valve calcinosis and fibrosis [21, 36–39]. Detection of at least one marker of extraosseous calcification may therefore provide a useful tool in everyday clinical practice.

The associations between biochemical parameters and BMD found in our study were largely as expected. The associations between BMD and tALP, iPTH and haemoglobin were found. In addition, tALP was positively associated with ABI. Interestingly, the associations between calcium and phosphate were significant at only one BMD site – the ribs. Similarly, to the results of Gorriz et al. study where VC degree was higher, the results of our study showed positive correlation between VC and phosphorus, PTH level in CKD patients [40].

The level of the new bone biomarker, plasma iFGF-23, was associated neither with VC nor with BMD. These results are similar to those of some previous studies [41] despite the apparently robust associations between iFGF-23 and progression of both AAC and arterial stiffness [11, 42, 43]. Definite conclusions should probably not yet be drawn.

This study must be interpreted with some limitations. There are relatively small number of participants. We used total body DEXA for assessment of BMD, but this method don`t able to divide long bones into portions for better measurement of BMD in cortical and trabecular sites. Another source of potential bias is cross-sectional design of this study. In spite of this fact, it is a good basis for future researches about vascular-bone axis, which can improve evidence about specific methods for detection, assessment and investigating relationship between bone and vasculature.

## Conclusions

Our work highlighted clinically important relationships between VC and bone mineral density (BMD) in CKD patients. We detected inverse relationships between AAC, high ABI and BMD. Secondly, BMD at certain bone sites (femur, femoral neck) and total BMD were associated with important lesions of heart valves. Thirdly, a significant association between a high ABI and lesions of the heart valves. The combined use of several non-invasive methods could probably allow better estimation of the presence and severity of VC, bone disease, and improve understanding of CKD-MBD. However, in our clinical routine, simple and non-invasive investigations can yield a great deal of information in individual patients when done repeatedly. Crucial mechanisms of the bone-vascular axis are not yet fully understood, and future studies of bone tissue microarchitecture and of the bone-vessel relationship are evidently still needed. We believe that the results of our study will help in the planning of future research and in current clinical practice for the early diagnosis, further monitoring and management of CKD-MBD. Additionally, these results may have treatment implications on use of different CKD-MBD medications.

## Data Availability

Research data are not publicly available on legal or ethical grounds. To request the data from this study please contact jana.uhlinova@kliinikum.ee.
